# Menthol as an Adjuvant to Help Athletes Cope With a Tropical Climate: Tracks From Heat Experiments With Special Focus on Guadeloupe Investigations

**DOI:** 10.3389/fphys.2019.01360

**Published:** 2019-10-31

**Authors:** Olivier Hue, Clovis Chabert, Aurélie Collado, Eric Hermand

**Affiliations:** Laboratoire ACTES, UPRES-EA 3596, UFR-STAPS, Université des Antilles, Guadeloupe, France

**Keywords:** long distance exercise, hot climate, hydration, thermoregulation, performance

## Abstract

Endurance and prolonged exercise are altered by hot climate. In hot and dry climate, thermoregulation processes, including evapotranspiration, normally maintain a relatively constant body core temperature. In hot and wet climate (usually called “tropical”), the decrease in evapotranspiration efficacy increases the sweating rate, which can rapidly induce severe hypohydration without efficiently reducing core temperature. The negative effects of tropical environment on long-duration exercise have been well documented, with clear demonstrations that they exceed the acclimation possibilities: both acclimated athletes and natives to tropical climate show impaired performances compared with that in neutral climate. New countermeasures, applicable during competitive events, are therefore needed to limit these negative effects. We studied the effects of several countermeasures in outdoor or natural tropical climates and noted that the easiest method to apply is cooling with cold (−1 to 3°C) beverage. Moreover, adding menthol increased the cold sensation induced by the beverage temperature, optimizing the positive effects on performance. We also demonstrated that efficient pre-cooling with cold menthol beverage requires drinking for 1 h instead of 30 min before the exercise. The optimal cooling method seems to be 1 h of cold + menthol pre-cooling ingestion followed by menthol + ice-slurry per-cooling. However, limitations should be noted: (1) the menthol concentration seems to be crucial, with positive effects for a 0.05% solution, whereas higher concentrations need to be explored; and (2) because it acts as a cold adjuvant without decreasing core temperature, menthol can lead to decreased thermoregulatory processes, thus inducing hyperthermia. Last, if menthol is added to cooling processes, athletes should first test them in training conditions for the maximal cooling effect to ensure optimal performance in competition in tropical climate.

## Introduction

The tropical climate is characterized by hot and wet conditions and has a negative impact on endurance exercise (Hue, 2011). Its deleterious effects have been demonstrated for swimming (Costill et al., 1967), cycling (Maughan et al., 2012), and running (Ely et al., 2007), and have been extrapolated to other cyclic and noncyclic exercise for durations exceeding 20 min (Hue, 2011). Its specificity is high humidity, which impedes efficient sweat evaporation. Indeed, humidity alone has an impact on performance by reducing thermolysis processes: it was shown that the higher the humidity, the more negatively performance is impacted (Maughan et al., 2012). Notably, when environmental evaporative capacity is limited, adherence to physiological mechanisms used in a hot and dry climate (reducing heat storage by increasing the sweating rate) creates a physiological disadvantage (Shapiro et al., 1998) that causes severe dehydration. The only way to achieve a match between heat gain and heat dissipation during outdoor exercise is by reducing the metabolic heat production, thus the exercise intensity. More than 33% of the world’s population lives in the tropics, and the 2014 Football World Cup and 2016 Olympic Games took place in this climate (both in Brazil), as will the 2020 Olympic Games in Tokyo where a mean 30°C and 80% rh are expected.

Many factors contribute to the decreased endurance performance in tropical conditions, although the exact causes are not well known. One of the most well-described factors is thermoregulation, which induces changes in cardiovascular processes (González-Alonso et al., 2008) and unbalances the hydration status by increasing the sweating rate. Furthermore, thermoregulation processes can decrease the blood volume dedicated to active muscles, which is accentuated by the total blood volume reduction linked to dehydration, further impairing performance. An anticipatory strategy may also be involved: in this case, the central nervous system (CNS) regulates the exercise intensity to limit excessive hyperthermia (Noakes, 2012). A critical core temperature was also proposed as a limiting factor in uncompensable heat stress (Nielsen et al., 1993).

## Acclimation Strategies and Cooling

Although heat adaptation has many benefits when the ambient temperature is high (Tyler et al., 2016), the adaptation to heat alone is insufficient to ensure similar performances in tropical compared to neutral climates (Voltaire et al., 2002; Hue et al., 2004). Moreover, athletes native to and living in tropical climates show decreased performances comparable to those living in neutral climates (Voltaire et al., 2003), demonstrating that environmental conditions surpass both physiological and psychological adaptations.

Strategies like pre-cooling and per-cooling protocols nevertheless minimize the impact of tropical climate on endurance performance with various degrees of success. Some, like cold-water immersion (Clements et al., 2002; Vaile et al., 2008) and cooling vests, have been studied but remain difficult to apply in real sports contexts (Ihsan et al., 2010; Hue, 2011; Siegel and Laursen, 2012). Pouring water is an efficient strategy in the heat (Morris and Jay, 2016), but the lower evaporative capacity in wet environments drastically reduces its benefits (Morris and Jay, 2016). However, the strategy of drinking cold-water/ice-slurry or cold-water/ice-slurry combined with menthol before or during exercise has been proven beneficial for endurance performance in the heat (Lee et al., 2008; Ihsan et al., 2010; Riera et al., 2014; Tran Trong et al., 2015; Best et al., 2018).

## Menthol as Adjuvant

Menthol is a compound of plant origin (Mentha) that specifically stimulates cold receptors in the skin and the mucous membranes of the oropharyngeal cavity, which are especially sensitive to menthol. When applied to skin and mucosal surfaces, menthol provokes a cooling sensation similar to the action of spraying cold-water on the face (Eccles et al., 1990). In the mouth, menthol is able to augment cold sensations and modulate taste-receptor activity (Green, 1985). One suggestion is that, by stimulating the major palatine nerve, oral menthol administration also directly influences the nasal sensation of airflow (Naito et al., 1997). Indeed, oral menthol administration by lozenge caused subjective sensations of improved airflow without actual changes in airway resistance in subjects suffering from nasal congestion (Eccles et al., 1990). Eccles et al. (1990) summarized these findings by noting that menthol can influence thirst, the drive to breathe, and arousal due to its effects on oral and nasal cold receptors. Thus, menthol use might be an additional asset to enhance exercise performance in hot climates (Mündel and Jones, 2010). Swilling an L(−) menthol solution was recently shown to increase exercise cycling time to exhaustion, suggesting that a change in oropharyngeal temperature perception during exercise in the heat positively affects endurance capacity (Mündel and Jones, 2010) and the perception of effort. Recently, Stevens and Best (2017) reported that “Menthol applied internally via a mouth rinse or a beverage containing menthol during endurance exercise in the heat is beneficial for performance.” Although menthol does not cool, it was hypothesized that its use with very cold or iced water reinforces cold sensations, enhancing performances in hot-wet or dry environments by decreasing both hot sensations and the rate of perceived exertion. Indeed, in their meta-analysis, Wegmann et al. (2012) reported that psychological effects, influenced by thermal sensations and ratings of perceived exertion, had a strong influence in warm environments.

## The Example of Guadeloupe Experiments

Because there are several other comprehensive reviews on this topic (Stevens and Best, 2017; Best et al., 2018), the purpose of the current mini-review was to focus on results from a (real) tropical environment: The Guadeloupe island. This chapter presents some of the results of studies using cold-water with or without menthol as an adjuvant during prolonged exercise in a tropical climate (Guadeloupe, France, Caribbean Islands). The objective is to define a strategy to help athletes cope with the negative effects of the tropical climate they will potentially encounter during their careers (e.g., Tokyo Olympic Games 2020). Guadeloupe is a good example of a tropical environment: the island is located in the Caribbean Sea in the northern hemisphere (16°N–60°W) and has a mean (daytime and nocturnal) temperature of 25–26°C and a mean relative humidity of 80–82%. The temperature is relatively constant, with about a 3.2°C difference between the hottest and coldest months (mean: 26.7°C in June–August; 23.5°C in January–February). The humidity, however, is quite variable, with a “humid” season (June–November, always >85% rh) and a “dry” one (December–May, mean: 75% rh).

The negative impact of Guadeloupe’s tropical climate on the running performance of young internationally ranked triathletes has been clearly demonstrated (Voltaire et al., 2002; Hue et al., 2004). These athletes had high VO_2_max, were young, and trained twice a day in the climate, three parameters known to facilitate the acclimation process (Lind and Bass, 1963; Pandolf et al., 1977). Yet 14 days of acclimation did not prevent performance from decreasing more than 7 days of acclimation did (compared with neutral climate), suggesting that aerobic performance is *de facto* negatively affected by tropical climate (Voltaire et al., 2002; Hue et al., 2004). This was corroborated in natives living in Guadeloupe (Voltaire et al., 2003): living and training for years in this tropical environment did not prevent its deleterious effects on aerobic performance. Therefore, strategies are needed to limit the negative effect of tropical climate during prolonged exercise.

Immersion in cold-water seems to be one of the more effective strategies. In their meta-analysis, Wegmann et al. (2012) reported that pre-exercise immersion provided the best performances in situations of high thermal stress (high ambient temperature and/or long-duration exercise; Duffield and Marino, 2007). This was supported by our findings that the performances in tropical climate of both elite and average-level paddlers (Hue et al., 2014) were related to the increase in core temperature and that pre-race and end-race core temperatures were positively correlated, reinforcing the importance of beginning competition with a lower body temperature. The relationship between core temperature increase and performance was partially corroborated during a long trail race (Baillot et al., 2014) in which the best performers finished with the higher core temperatures. However, because the pre-cooling effect seemed to decrease rapidly, it was hypothesized that the effect is limited when exercise lasts over 60 min (Wegmann et al., 2012). For shorter exercise durations, pre-exercise water immersion was more effective than active recovery in maintaining subsequent high-intensity cycling performance (Vaile et al., 2008).

Interestingly, we recently demonstrated that adding menthol to water enhances the benefits of a 10-min immersion in cold-water between two cycling bouts (Rinaldi et al., 2018), confirming the positive effect of menthol as an adjuvant during exercise in tropical climate.

Our focus on adding menthol to cold/iced water began with the results of a study of a 27-km trail race in tropical climate. In this work, we clearly demonstrated that reaching a critical core temperature and the dehydration status were not the major parameters of the performance limitation in this climate. Indeed, we noted that the greater the dehydration (calculated as total body water loss) and esophageal temperature, the better performance was in 20 well-trained runners, living and training in tropical climate (Baillot et al., 2014). We concomitantly demonstrated that the fastest runners reported lower thermal sensations despite higher core temperatures, whereas the slowest runners reported higher thermal sensations with lower core temperatures. These results suggested that thermal sensation, more than dehydration and hyperthermia, positively influences performance in tropical climate (Baillot et al., 2014). In a laboratory study, we confirmed the positive influence of drinking 190 ml of a beverage combining menthol (as opposed to non-menthol) and cold-water or ice-slurry every 5 km during a 20-km all-out cycling test in 30.7°C–78% rh conditions. The cyclists performed better with the menthol beverages: 163 s saved with menthol + cold-water and 233 s with menthol + ice-slurry; respectively, −7.2 and −10.3% of the performance with neutral/non-menthol beverage (Riera et al., 2014). These results were corroborated during outdoor tests when 10 trained male triathletes performed five blocks consisting of 4-km cycling and 1.5-km running in 32.5°C-57% rh, drinking 190 ml of neutral, cold or iced beverages with menthol after each cycle-run block. Global performance was better with menthol + ice-slurry (−289 s; −6.2%) than menthol + cold-water (−136 s; −3%) or menthol + neutral-water (Tran Trong et al., 2015). In both experiments, the positive effect of menthol + ice-slurry was significantly more pronounced during the last third of the race, accompanied by significantly decreased thermal sensations or no increase in RPE despite a faster pace. A plausible hypothesis for these results with ice-slurry is that ingesting menthol + ice-slurry created more pleasant sensations and/or an incorrect assessment of thermal stress, enabled an increase in exercise performance.

These results demonstrated that menthol + cold-water beverages are useful during aerobic competition in tropical climate. However, as noted above, it seems important to start the competition with a low core temperature (Hue et al., 2015). Bongers et al. (2015) suggested that combining pre-cooling and per-cooling might be more effective to improve exercise performance than a single cooling strategy. Therefore, we explored this hypothesis in a 30-km cycling trial in tropical climate by adding 30 min of pre-cooling, drinking cold-water (3°C) versus neutral-water (23°C) beverage (i.e., 7 g.kg^−1^ at 0, 15, and 30 min of exercise) to per-cooling with 7 g.kg^−1^ of menthol + ice-slurry (−1°C) beverage drank every 7.5 km of cycling (Riera et al., 2016). Despite no change in performance, core temperatures with cold beverage were lower at the end of pre-cooling (a drop of 0.3°C that disappeared at the fifth km) and RPE was decreased in the last stage of the 30 km. We suggested that 30 min of pre-cooling with cold-water before an ice-slurry-percooled exercise would be useful for longer exercises than our experimental trial and that a longer pre-cooling period (thus more cold fluid ingestion before exercise) would certainly provide further benefits to subsequent exercise. Furthermore, the plasma half-life of menthol being 56.2 min when taken in a capsule containing 100 mg of menthol (Gelal et al., 1999), we might expect that longer pre-cooling with menthol ingestion would potentiate the menthol concentration in the brain. Therefore, studies with a longer pre-cooling period have to be investigated.

Recently, some studies have focused on spraying or pouring water (or cold-water with or without menthol) during exercise in tropical climate. Indeed, spraying or pouring water over the face and/or body can improve performance in tropical conditions. For example, pouring cold-water over the skin will reduce skin temperature before dripping off the body and transiently improve thermal comfort. Cooling the head in this manner resulted in a 51% increase in cycling time to exhaustion at 75% VO_2max_ (Ansley et al., 2008), with similar effects recently observed in running with intermittent water (22°C) spray (Stevens et al., 2017). Neck cooling during a 90-min running trial in a hot environment (30.4°C and 53% rh) increased the covered distance by 7.4% with no change in rectal temperature (Tyler and Sunderland, 2011). However, the positive effects of these methods might be attenuated by the exercise duration. Indeed, when spraying or pouring in an uncompensated thermal stress environment, the liquid is rapidly evacuated from the skin surface to the floor by sweat, thus have a lower cooling effect (Morris and Jay, 2016). Moreover, the efficiency of these techniques is mainly due to the evaporative processes, which are dramatically decreased in a humid environment, and they thus have to be explored in tropical climate. It has been demonstrated that the degree of cooling sensation in a body area caused by menthol correlates inversely with a thicker stratum corneum, as a thicker stratum corneum is a more difficult barrier to penetrate (Watson et al., 1978). One might thus suspect that, for the same menthol dose, the tongue and oral cavity are more sensitive to menthol in comparison with other body parts (Watson et al., 1978).

Most studies on the effects of cutaneous menthol are based on the use of a 0.05% solution. Because a higher concentration has been demonstrated to have a positive effect on time-limited performance (Schlader et al., 2011), it should be investigated in the future.

Last, in a recent review on topical and ingested cooling methodologies for endurance exercise performance in the heat, Best et al. (2018) reported that if topical or combined methods improved physiological or perceptual responses, ingested cooling (and ingestion of menthol) could also positively increased athletes’ time-trial performances.

## Limitations

As noted in previous chapters, menthol use and, even more, combined menthol + cold-water/ice-slurry beverages for pre- or per-cooling may be efficient adjuvants to limit the negative effects of tropical climate on performance. However, some limitations should be noted, as these effects are directly due to the menthol mechanism. Menthol acts on the TRPM8, TRPA1, and TRPV3 thermoreceptors (Macpherson et al., 2006), hence modifying thermal perceptions of the body. This thermal-sensitive effect of menthol is concentration-dependent and can lead to colder or warmer sensations (Macpherson et al., 2006). At a 0.05% concentration, the most frequently used dosage, menthol drinking induces cold sensations (Riera et al., 2014). When used as a spray or poured on the skin, a 0.05% menthol solution also induces cold sensations (Gillis et al., 2010). But at 2%, it can have a vasoconstrictor effect, lowering the sweating response and thus increasing the core temperature (Gillis et al., 2010). However, it was also reported that a higher concentration (i.e., 8%) can have positive effects on time-limited performance (Schlader et al., 2011) and potentially increase body heat dissipation by higher peripheral vasodilatation (Craighead and Alexander, 2016).

The link shown between menthol and the GABAa neurotransmitters, which decrease neuronal activity (Hall et al., 2004), suggests that menthol, when fixed on GABAa receptors, limits thermal stress perception and certainly distorts the thermal information sent to the brain. This distortion, by decreasing thermal stress and thus RPE (as noted in some of our studies), enables better performances when the limitation is due to environmental sensation. Yet, by providing misleading thermal information, menthol induces decreases in the thermoregulation processes, which can then increase core temperature, although this can be partially corrected by drinking very cold or iced water. Nevertheless, we cannot exclude the possibility that menthol use might cause hyperthermia in some athletes due to a failure in the thermoregulation processes, particularly in most successful athletes with higher thermal limits. The critical core temperature is dependent on factors (González-Alonso et al., 2008) like age, sex, hydration status, and most importantly training status and motivation. Baillot et al. (2014) demonstrated that better trailers finished with higher water losses and core temperatures, and this was corroborated in high-level paddlers (Hue et al., 2014). Impaired thermoregulation processes after menthol + water immersion were noted by Rinaldi et al. (2018).

## Conclusions and Practical Implications

To help athletes cope with the negative effects of the tropical climate they will likely encounter in Tokyo 2020, we have summarized our findings with the following recommendations:

1. When possible, use pre-cooling immersion if the subsequent exercise does not last more than 30 min; adding menthol improves performance more than non-menthol immersion. Add per-cooling as described in 3.
2. When immersion is not possible, use pre-cooling with beverage: the colder the drink, the better the effects. The use of 0.05% menthol will increase the cold sensations induced by beverage ingestion. Add 3.
3. Adding per-cooling with very cold (−1 to 3°C) beverages may be useful for long-duration events (more than 1 h). Adding 0.05% menthol appears to potentiate the benefits if it is well tolerated by the athlete.
4. When possible, pour (very) cold water during exercise (cycling, running, walking, or any sport permitting pouring) as often as possible.

Each athlete nevertheless has a specific sensitivity and physiological response to these countermeasures, and menthol might act as a severe vasoconstrictor for some, limiting the thermoregulation processes and thus increasing core temperature. We therefore strongly recommend testing these methods before race day to optimize their benefits and avoid potential adverse outcomes such as gastric disturbance.

## Author Contributions

All authors have contributed to the manuscript, in data collection, data analysis or the write of the manuscript.

### Conflict of Interest

The authors declare that the research was conducted in the absence of any commercial or financial relationships that could be construed as a potential conflict of interest.

## References

[ref1] Ansley L. Marvin G. Sharma A. Kendall M. J. Jones D. A. Bridge M. W. (2008). The effects of head cooling on endurance and neuroendocrine responses to exercise in warm conditions. Physiol. Res. 57, 863–872. 18052690

[ref2] Baillot M. Le Bris S. Hue O. (2014). Fluid replacement strategy during a 27-km trail run in hot and humid conditions. Int. J. Sports Med. 35, 147–152. 10.1055/s-0033-1349108 23868683

[ref3] Best R. Payton S. Spears I. Riera F. Berger N. (2018). Topical and ingested cooling methodologies for endurance exercise performance in the heat. Sports 6, pii: E11. 10.3390/sports6010011 29910315 PMC5969198

[ref4] Bongers C. C. W. G. Thijssen D. H. J. Veltmeijer M. T. W. Hopman M. T. E. Eijsvogels T. M. H. (2015). Precooling and percooling (cooling during exercise) both improve performance in the heat: a meta-analytical review. Br. J. Sports Med. 49, 377–384. 10.1136/bjsports-2013-092928 24747298

[ref5] Clements J. M. Casa D. J. Knight J. McClung J. M. Blake A. S. Meenen P. M. (2002). Ice-water immersion and cold-water immersion provide similar cooling rates in runners with exercise-induced hyperthermia. J. Athl. Train. 37, 146–150. 12937427 PMC164337

[ref6] Costill D. L. Cahill P. J. Eddy D. (1967). Metabolic responses to submaximal exercise in three water temperatures. J. Appl. Physiol. 22, 628–632. 10.1152/jappl.1967.22.4.628 6023173

[ref7] Craighead D. H. Alexander L. M. (2016). Topical menthol increases cutaneous blood flow. Microvasc. Res. 107, 39–45. 10.1016/j.mvr.2016.04.010 27131832 PMC5406845

[ref8] Duffield R. Marino F. E. (2007). Effects of pre-cooling procedures on intermittent-sprint exercise performance in warm conditions. Eur. J. Appl. Physiol. 100, 727–735. 10.1007/s00421-007-0468-x 17476523

[ref9] Eccles R. Jawad M. S. Morris S. (1990). The effects of oral administration of (−)-menthol on nasal resistance to airflow and nasal sensation of airflow in subjects suffering from nasal congestion associated with the common cold. J. Pharm. Pharmacol. 42, 652–654. 1981905 10.1111/j.2042-7158.1990.tb06625.x

[ref10] Ely M. R. Cheuvront S. N. Roberts W. O. Montain S. J. (2007). Impact of weather on marathon-running performance. Med. Sci. Sports Exerc. 39, 487–493. 10.1249/mss.0b013e31802d3aba 17473775

[ref11] Gelal A. Jacob P. Yu L. Benowitz N. L. (1999). Disposition kinetics and effects of menthol. Clin. Pharmacol. Ther. 66, 128–135. 10.1053/cp.1999.v66.100455001 10460066

[ref12] Gillis D. J. House J. R. Tipton M. J. (2010). The influence of menthol on thermoregulation and perception during exercise in warm, humid conditions. Eur. J. Appl. Physiol. 110, 609–618. 10.1007/s00421-010-1533-4 20574677

[ref13] González-Alonso J. Crandall C. G. Johnson J. M. (2008). The cardiovascular challenge of exercising in the heat. J. Physiol. 586, 45–53. 10.1113/jphysiol.2007.142158 17855754 PMC2375553

[ref14] Green B. G. (1985). Menthol modulates oral sensations of warmth and cold. Physiol. Behav. 35, 427–434. 4070414 10.1016/0031-9384(85)90319-1

[ref15] Hall A. C. Turcotte C. M. Betts B. A. Yeung W.-Y. Agyeman A. S. Burk L. A. (2004). Modulation of human GABAA and glycine receptor currents by menthol and related monoterpenoids. Eur. J. Pharmacol. 506, 9–16. 10.1016/j.ejphar.2004.10.026 15588619

[ref16] Hue O. (2011). The challenge of performing aerobic exercise in tropical environments: applied knowledge and perspectives. Int. J. Sports Physiol. Perform. 6, 443–454. 22248546 10.1123/ijspp.6.4.443

[ref17] Hue O. Le Jeannic P. Chamari K. (2014). A pilot study on how do elite surfski padllers manage their effort and hydration pattern in the heat. Biol. Sport 31, 283–288. 10.5604/20831862.1120936 25435671 PMC4203845

[ref18] Hue O. Le Jeannic P. Chamari K. (2015). Self-hydration and thermoregulatory processes of average-level paddlers during international surfski events in a tropical climate. Biol. Sport 32, 329–332. 10.5604/20831862.1188610 28479662 PMC5394853

[ref19] Hue O. Voltaire B. Galy O. Costes O. Callis A. Hertogh C. (2004). Effects of 8 days acclimation on biological and performance response in a tropical climate. J. Sports Med. Phys. Fitness 44, 30–37. 15181387

[ref20] Ihsan M. Landers G. Brearley M. Peeling P. (2010). Beneficial effects of ice ingestion as a precooling strategy on 40-km cycling time-trial performance. Int. J. Sports Physiol. Perform. 5, 140–151. 20625187 10.1123/ijspp.5.2.140

[ref21] Lee J. K. W. Shirreffs S. M. Maughan R. J. (2008). Cold drink ingestion improves exercise endurance capacity in the heat. Med. Sci. Sports Exerc. 40, 1637–1644. 10.1249/MSS.0b013e318178465d 18685527

[ref22] Lind A. R. Bass D. E. (1963). Optimal exposure time for development of acclimatization to heat. Fed. Proc. 22, 704–708. 13930724

[ref23] Macpherson L. J. Hwang S. W. Miyamoto T. Dubin A. E. Patapoutian A. Story G. M. (2006). More than cool: promiscuous relationships of menthol and other sensory compounds. Mol. Cell. Neurosci. 32, 335–343. 10.1016/j.mcn.2006.05.005 16829128

[ref24] Maughan R. J. Otani H. Watson P. (2012). Influence of relative humidity on prolonged exercise capacity in a warm environment. Eur. J. Appl. Physiol. 112, 2313–2321. 10.1007/s00421-011-2206-7 22012542

[ref25] Morris N. B. Jay O. (2016). To drink or to pour: how should athletes use water to cool themselves? Temperature 3, 191–194. 10.1080/23328940.2016.1185206 PMC4964991 27857948

[ref26] Mündel T. Jones D. A. (2010). The effects of swilling an L(−)-menthol solution during exercise in the heat. Eur. J. Appl. Physiol. 109, 59–65. 10.1007/s00421-009-1180-9 19727797

[ref27] Naito K. Komori M. Kondo Y. Takeuchi M. Iwata S. (1997). The effect of L-menthol stimulation of the major palatine nerve on subjective and objective nasal patency. Auris Nasus Larynx 24, 159–162. 10.1016/S0385-8146(96)00005-3 9134138

[ref28] Nielsen B. Hales J. R. Strange S. Christensen N. J. Warberg J. Saltin B. (1993). Human circulatory and thermoregulatory adaptations with heat acclimation and exercise in a hot, dry environment. J. Physiol. 460, 467–485. 8487204 10.1113/jphysiol.1993.sp019482 PMC1175224

[ref29] Noakes T. D. (2012). Fatigue is a brain-derived emotion that regulates the exercise behavior to ensure the protection of whole body homeostasis. Front. Physiol. 3:82. 10.3389/fphys.2012.00082 22514538 PMC3323922

[ref30] Pandolf K. B. Burse R. L. Goldman R. F. (1977). Role of physical fitness in heat acclimatisation, decay and reinduction. Ergonomics 20, 399–408. 10.1080/00140137708931642 908323

[ref31] Riera F. Trong T. T. Rinaldi K. Hue O. (2016). Precooling does not enhance the effect on performance of midcooling with ice-slush/menthol. Int. J. Sports Med. 37, 1025–1031. 10.1055/s-0042-107597 27706550

[ref32] Riera F. Trong T. T. Sinnapah S. Hue O. (2014). Physical and perceptual cooling with beverages to increase cycle performance in a tropical climate. PLoS One 9:e103718. 10.1371/journal.pone.0103718 25084009 PMC4118924

[ref33] Rinaldi K. Trong T. T. Riera F. Appel K. Hue O. (2018). Immersion with menthol improves recovery between 2 cycling exercises in hot and humid environment. Appl. Physiol. Nutr. Metab. 43, 902–908. 10.1139/apnm-2017-0525 29533726

[ref34] Schlader Z. J. Simmons S. E. Stannard S. R. Mündel T. (2011). The independent roles of temperature and thermal perception in the control of human thermoregulatory behavior. Physiol. Behav. 103, 217–224. 10.1016/j.physbeh.2011.02.002 21315099

[ref35] Shapiro Y. Moran D. Epstein Y. (1998). Acclimatization strategies–preparing for exercise in the heat. Int. J. Sports Med. 19(Suppl. 2), S161–S163. 10.1055/s-2007-971986 9694427

[ref36] Siegel R. Laursen P. B. (2012). Keeping your cool: possible mechanisms for enhanced exercise performance in the heat with internal cooling methods. Sports Med. 42, 89–98. 10.2165/11596870-000000000-00000 22175533

[ref37] Stevens C. J. Best R. (2017). Menthol: a fresh ergogenic aid for athletic performance. Sports Med. 47, 1035–1042. 10.1007/s40279-016-0652-4 27858306

[ref38] Stevens C. J. Kittel A. Sculley D. V. Callister R. Taylor L. Dascombe B. J. (2017). Running performance in the heat is improved by similar magnitude with pre-exercise cold-water immersion and mid-exercise facial water spray. J. Sports Sci. 35, 798–805. 10.1080/02640414.2016.1192294 27267974

[ref39] Tran Trong T. Riera F. Rinaldi K. Briki W. Hue O. (2015). Ingestion of a cold temperature/menthol beverage increases outdoor exercise performance in a hot, humid environment. PLoS One 10:e0123815. 10.1371/journal.pone.0123815 25856401 PMC4391868

[ref40] Tyler C. J. Reeve T. Hodges G. J. Cheung S. S. (2016). The effects of heat adaptation on physiology, perception and exercise performance in the heat: a meta-analysis. Sports Med. 46, 1699–1724. 10.1007/s40279-016-0538-5 27106556

[ref41] Tyler C. J. Sunderland C. (2011). Neck cooling and running performance in the heat: single versus repeated application. Med. Sci. Sports Exerc. 43, 2388–2395. 10.1249/MSS.0b013e318222ef72 21606877

[ref42] Vaile J. Halson S. Gill N. Dawson B. (2008). Effect of cold water immersion on repeat cycling performance and thermoregulation in the heat. J. Sports Sci. 26, 431–440. 10.1080/02640410701567425 18274940

[ref43] Voltaire B. Berthouze-Aranda S. Hue O. (2003). Influence of a hot/wet environment on exercise performance in natives to tropical climate. J. Sports Med. Phys. Fitness 43, 306–311. 14625511

[ref44] Voltaire B. Galy O. Coste O. Racinais S. Callis A. Blonc S. (2002). Effect of fourteen days of acclimatization on athletic performance in tropical climate. Can. J. Appl. Physiol. 27, 551–562. 10.1139/h02-031 12500994

[ref45] Watson H. R. Hems R. Rowsell D. G. Spring D. J. (1978). New compounds with the menthol cooling effect. J. Soc. Cosmet. Chem. 29, 185–200.

[ref46] Wegmann M. Faude O. Poppendieck W. Hecksteden A. Fröhlich M. Meyer T. (2012). Pre-cooling and sports performance: a meta-analytical review. Sports Med. 42, 545–564. 10.2165/11630550-000000000-00000 22642829

